# Atypical survey response patterns as early indicators of dementia onset: a coordinated analysis across longitudinal aging cohorts

**DOI:** 10.1093/geronb/gbag197

**Published:** 2026-09-18

**Authors:** Stefan Schneider, Pey-Jiuan Lee, Doerte U Junghaenel, Erik Meijer, Raymond Hernandez, Haomiao Jin, Arthur A Stone, Arie Kapteyn, Bart Orriens, Hongxin Gao, Elizabeth M Zelinski

**Affiliations:** Center for Economic and Social Research, University of Southern California, Los Angeles, California, United States; Department of Psychology, University of Southern California, Los Angeles, California, United States; Center for Economic and Social Research, University of Southern California, Los Angeles, California, United States; Center for Economic and Social Research, University of Southern California, Los Angeles, California, United States; Department of Psychology, University of Southern California, Los Angeles, California, United States; Center for Economic and Social Research, University of Southern California, Los Angeles, California, United States; Center for Economic and Social Research, University of Southern California, Los Angeles, California, United States; School of Health Sciences, Faculty of Health and Medical Sciences, University of Surrey, Guildford, United Kingdom; Center for Economic and Social Research, University of Southern California, Los Angeles, California, United States; Department of Psychology, University of Southern California, Los Angeles, California, United States; Center for Economic and Social Research, University of Southern California, Los Angeles, California, United States; Center for Economic and Social Research, University of Southern California, Los Angeles, California, United States; School of Health Sciences, Faculty of Health and Medical Sciences, University of Surrey, Guildford, United Kingdom; Leonard Davis School of Gerontology, University of Southern California, Los Angeles, California, United States

**Keywords:** Survey response behaviors, Response inconsistency, Person-fit statistics, Longitudinal cohorts, Survival analysis

## Abstract

**Objectives:**

Atypical survey response patterns, such as inconsistent or implausible responses, have been linked to lower cognitive functioning in later life. This study examined whether these behaviors predict future dementia onset across diverse longitudinal studies of aging.

**Methods:**

We conducted a coordinated analysis of data from eight longitudinal studies of aging (total *N *= 76,350). We derived 5 common types of atypical response pattern indicators from participants’ questionnaire data. Associations with subsequent dementia risk were examined within each cohort using Cox proportional hazards models adjusted for demographic covariates and accounting for death as a competing risk. Results were synthesized using random-effects meta-analysis methods.

**Results:**

Across cohorts, significant associations with incident dementia were observed for item-nonresponse (overall hazard ratio [HR] per *SD* increase = 1.06), random response errors (HR = 1.25), multivariate outlier responses (HR = 1.31), and incompatible response patterns indexed by Guttman errors and person-fit statistics (HRs = 1.22–1.25). Extreme responding was not significantly associated with dementia risk. Secondary analyses suggested stronger associations among individuals younger than 75 years and those with higher levels of education.

**Discussion:**

Routine survey responses contain behavioral signals associated with emerging dementia risk. Because these indicators can be derived from existing survey data without additional testing burden, they may provide a scalable complement to traditional approaches for studying cognitive aging in large population-based cohorts.

Cognitive decline associated with dementia often develops gradually over many years before clinical diagnosis. During this period, subtle changes in everyday behavior may emerge well before dementia is formally recognized (Verlinden et al., 2016). Identifying subtle behavioral indicators associated with increased dementia risk is therefore an important challenge for research on cognitive aging.

Longitudinal studies of aging routinely collect extensive questionnaire data as part of broader assessments of health, functioning, and cognition. Although questionnaires are primarily designed to obtain self-reported information, completing them is also a standardized behavioral activity. Survey response behaviors may therefore provide an additional, largely unexamined source of information relevant to cognitive aging.

Completing a survey places demands on multiple cognitive processes, including reading comprehension, working memory, numerical reasoning, and the ability to apply response formats consistently (Tourangeau et al., 2000). Survey methodology research has long documented that some respondents exhibit skipped items, erratic responses, implausible response combinations, or unusually extreme response patterns. These behaviors represent partially overlapping but conceptually distinguishable departures from typical survey responding, ranging from omissions and local inconsistency to unusual response profiles, incompatible within-scale patterns, and general response-style tendencies. They are usually viewed as threats to data quality. From a cognitive aging perspective, however, they may also reflect meaningful variation in the cognitive processes involved in understanding questions, selecting responses, and maintaining consistency across related items (Jin et al., 2023; Knäuper et al., 2016; Schneider, Hernandez, et al., 2024).

Recent work has shown that survey response patterns covary systematically with cognitive abilities in later life. Across multiple longitudinal studies of aging, older adults with lower cognitive test scores tend to produce more inconsistent, implausible, or error-prone survey responses (Schneider, Lee, et al., 2024). These findings suggest that survey response patterns are not merely nuisance variation in self-report data but may function as behavioral indicators relevant to cognitive functioning. Because such indicators can be derived from existing survey data, they may help characterize cognitive functioning in large population-based studies without adding new assessment burden. The primary value of such indicators is for research applications, where they can add cognitive-relevant behavioral information to longitudinal cohorts that already collect extensive questionnaire data.

Prospective evidence linking survey response behaviors to cognitive decline and dementia risk remains limited. To our knowledge, only one published prospective study has examined whether a range of atypical response patterns in self-report questionnaires predicts incident dementia. In the Health and Retirement Study (HRS), older adults who exhibited more problematic response patterns were at elevated risk of developing dementia over subsequent years (Schneider et al., 2021). Whether this association generalizes beyond a single study is unknown. Aging cohorts differ substantially in survey content, languages, populations, and approaches to dementia ascertainment, raising the possibility that previously observed associations reflect study-specific features rather than a robust signal of cognitive decline.

The present study aimed to evaluate the generalizability of atypical survey response patterns as early markers of dementia onset across multiple longitudinal studies of aging. Using data from eight large-scale panel studies, we derived a common set of response pattern indicators from raw survey data and examined whether they predicted subsequent dementia within each cohort, accounting for death as a competing risk. We integrated results within a coordinated analysis framework (Curran & Hussong, 2009) using individual-participant-data meta-analysis techniques (Clegg et al., 2022), which allows effect sizes to be estimated separately within each study and synthesized across studies while preserving heterogeneity. This approach provides a stringent test of whether survey response patterns capture cross-cohort signals of emerging cognitive impairment.

## Method

### Study samples

The following studies were included (for details, see Supplementary Table 1.1–1.8): Australian Longitudinal Study of Ageing (ALSA; Luszcz et al., 2016), Canberra Longitudinal Study (CLS; Christensen et al., 2004), Einstein Aging Study (EAS; Katz et al., 2012), English Longitudinal Study of Ageing (ELSA; Steptoe et al., 2013), HRS (Sonnega et al., 2014), Swedish Adoption Twin Study of Aging (SATSA; Pedersen et al., 1991), Survey of Health, Ageing and Retirement in Europe (SHARE; Börsch-Supan et al., 2013), and The Irish Longitudinal Study on Ageing (TILDA; Whelan & Savva, 2013). We included participants aged 50 or older who provided questionnaire data to derive survey response patterns and had at least one follow-up assessment of dementia status. Participants classified as having dementia at their first assessment wave were excluded. This research was approved by the University of Southern California’s Institutional Review Board. All participants provided informed consent as part of the studies from which the data were drawn.

### Survey response pattern indicators

Five families of survey response pattern indices were derived in each study: (a) item-nonresponse, defined as the proportion of items skipped within each survey scale; (b) random response errors, reflecting erratic or locally inconsistent responses within a scale; (c) multivariate outlier responses, reflecting response profiles that were unusual relative to other respondents; (d) incompatible responses, assessed using Guttman errors and the person-fit statistics *lz* and U3, which quantify departures from the expected response structure within a scale; and (e) extreme responding, reflecting a tendency to select response-scale endpoints. Indices were derived from each respondent’s first available assessment wave. Supplementary Table 2 provides computational details.

All indices were computed exclusively from multi-item rating scales assessing a common underlying construct (e.g., personality traits, quality of life, subjective wellbeing). This restriction was important because several indices (e.g., random response errors, Guttman errors) rely on the expected consistency and structure of responses across conceptually related items. Specifically, analyses were restricted to scales with at least three items and ordinal response options. Reverse-keyed items were recoded before response pattern indices were calculated. Questions administered only to subsets of respondents (e.g., due to skip patterns) were excluded (for a list of included scales, see Supplementary Table 1).

### Dementia ascertainment

Details on how dementia was ascertained in each study are shown in Table 1. For each cohort, we followed established approaches used in prior studies of that dataset. Two studies used established cutoffs on cognitive test batteries together with informant reports (CLS, HRS), four studies used cutoffs on cognitive tests only (ALSA, EAS, SATSA, TILDA), and two studies relied on self-reported dementia diagnoses together with informant reports (ELSA, SHARE). Respondents were followed across all waves after their initial assessment used to derive the response pattern indices. Incident dementia was defined as the first wave meeting dementia criteria and was treated as a nonreversible (absorbing) state thereafter.

**Table 1:** Descriptive study characteristics.

Variables	ALSA	CLS	EAS	ELSA	HRS	SATSA	SHARE	TILDA
**Sample size**	1,420	600	1,114	12,913	14,596	1,257	38,376	6,074
**Sex: Male**	707 (49.79%)	285 (47.50%)	427 (38.33%)	5,977 (46.29%)	5,998 (41.09%)	494 (39.30%)	16,521 (43.05%)	2,736 (45.04%)
**Education level**								
Primary	909 (64.01%)	14 (2.33%)	62 (5.57%)	4,725 (36.59%)	2,177 (14.92%)	798 (63.48%)	14,066 (36.65%)	1,528 (25.16%)
Secondary	— ^a^	393 (65.50%)	398 (35.73%)	2,390 (18.51%)	5,123 (35.10%)	319 (25.38%)	16,219 (42.26%)	3,576 (58.87%)
Tertiary	508 (35.77%)	192 (32.00%)	654 (58.71%)	4,635 (35.89%)	7,294 (49.97%)	48 (3.82%)	8,091 (21.08%)	969 (15.95%)
Education unavailable	3 (0.21%)	1 (0.17%)	0 (0.00%)	1,163 (9.01%)	2 (0.01%)	92 (7.32%)	0 (0.00%)	1 (0.02%)
**Age at baseline**								
Age (years)	65–103	70–98	70–100	50–90	50–96	50–91	50–100	50–84
Mean age, years (*SD*)	77.16 (6.27)	76.35 (5.07)	77.75 (5.33)	62.00 (9.23)	64.70 (9.39)	65.46 (8.44)	63.86 (9.09)	62.93 (8.74)
Aged 75 years and older	877 (61.76%)	328 (54.67%)	732 (65.71%)	1,504 (11.65%)	2,476 (16.96%)	193 (15.35%)	5,575 (14.53%)	797 (13.12%)
**Max. number of follow-up waves**	9	3	17	8	7	5	7	3
**Max. follow-up duration (years)**	22	12	17	16	12	23	14	6
**Median follow-up duration (years)**	2	8	3	10	8	9	6	6
**Dementia incidence during follow-up**	19.37%	39.00%	19.57%	1.38%	7.16%	5.25%	3.06%	3.98%
**Dementia ascertainment method**	MMSE	MMSE, IQCODE	BIMC, FCSRT	Self-report, IQCODE	Langa-Weir (TICS and proxy)	MMSE	Self- and proxy-report	MMSE
**Observed mortality rate**	52.39%	17.00%	10.50%	2.88%	10.11%	23.07%	6.86%	— ^b^
**Survey scales used to calculate response-pattern indices, mean (range)**	15 (15–15)	12 (12–12)	5 (5–5)	9.8 (8–18)	16.09 (9–21)	24.12 (24–27)	9.15 (4–10)	5.53 (5–18)

*Note*. ALSA = Australian Longitudinal Study of Ageing; BIMC = Blessed Information-Memory-Concentration; CLS = Canberra Longitudinal Study; EAS = Einstein Aging Study; ELSA = English Longitudinal Study of Ageing; FCSRT = Free and Cued Selective Reminding Test; HRS = Health and Retirement Study; IQCODE = Informant Questionnaire on Cognitive Decline in the Elderly; MMSE = Mini-Mental Status Exam; SATSA = Swedish Adoption/Twin Study of Aging; *SD* = standard deviation**;** SHARE = Survey of Health, Ageing and Retirement in Europe; TICS = Telephone Interview of Cognitive Status; TILDA = The Irish Longitudinal Study on Ageing.

a Primary and secondary education levels were not distinguished in this study.

b Mortality data not available. Dementia percentages reflect observed cumulative dementia classifications within each analytic sample after exclusion of participants classified with dementia at baseline. These values are not directly comparable across cohorts because studies differed in baseline age, follow-up duration, attrition, and dementia ascertainment procedures.

### Data analysis

We used Cox proportional hazards regression models (PROC PHREG, SAS 9.4; Cary, NC) to examine associations between baseline survey response patterns (independent variables, analyzed in separate models) and subsequent incident dementia (dependent variable). Participants without dementia were censored at their last available dementia assessment unless death was observed first and modeled as a competing event. Survey response pattern indices were standardized prior to analysis so that hazard ratios (HRs) reflected the association with a one standard deviation increase in each indicator, facilitating comparison across indices. Model 1 was adjusted for age and sex, and Model 2 additionally adjusted for education. In Model 3, we further accounted for death as a competing event using Fine and Gray’s (1999) proportional subdistribution hazards approach in all studies with available mortality data (all except TILDA). In Model 4, we additionally adjusted for individuals’ study-specific baseline cognitive test scores (see Supplementary Table 3) to evaluate whether survey response-pattern indicators were incrementally associated with incident dementia beyond standard cognitive measures. The proportional-hazards assumption was evaluated within each study using covariate-by-log-time interaction tests for sex, age, education, and response-pattern indicators, with no consistent evidence of violations (Supplementary Table 4).

In secondary analyses, age (<75 vs ≥75 years), sex, and education (secondary and tertiary vs primary) were evaluated as potential moderators by testing their interactions with response patterns in Model 3. HRs from these models are reported as relative HRs (i.e., interaction effects), reflecting subgroup differences in associations between response patterns and dementia risk.

To aggregate results across studies, random-effects meta-analysis models were estimated separately for each response pattern index. HRs served as effect sizes. The *Q* statistic was used to test for effect size heterogeneity, and *I*² and τ^2^ were used to quantify its magnitude (Higgins et al., 2003). All meta-analyses were conducted using the *metafor* package (version 4.8.0) in R (version 4.2.1) with restricted maximum likelihood estimation (Viechtbauer, 2010).

## Results

Descriptive characteristics of the study samples are shown in Table 1. Overall, 4,979,890 survey item responses from 76,350 respondents were analyzed, including nonresponses. The average skipped-item rate was 3.3%. Study sample sizes ranged from 600 (CLS) to 38,376 (SHARE); 38% to 50% of participants were male; mean baseline age ranged from 62.00 years (ELSA) to 77.75 years (EAS); and maximum follow-up ranged from 6 years (TILDA) to 23 years (SATSA). Survey scales used to derive the indices ranged from 4 (SHARE) to 27 (SATSA). Observed dementia classifications ranged from 1.38% (ELSA) to 39.00% (CLS), likely reflecting differences in sample age, follow-up duration, and dementia ascertainment. These values describe the present analytic cohorts and should not be interpreted as directly comparable epidemiologic incidence or prevalence estimates.

### Primary analyses: associations between survey response patterns and incident dementia

Several indicators reflecting atypical survey response patterns were associated with elevated risk of incident dementia across studies (Table 2; Figure 1). Associations were somewhat attenuated after adjustment for education and competing mortality risk, but the overall pattern of findings remained similar across models. In the fully adjusted models (Model 3), greater item non-response (HR = 1.06, 95% confidence interval [CI] [1.01, 1.12]), more random response errors (HR = 1.25, 95% CI [1.12, 1.40]), multivariate outlier responses (HR = 1.31, 95% CI [1.16, 1.49]), and incompatible responses assessed via Guttman errors (HR = 1.25, 95% CI [1.15, 1.37]) were associated with elevated dementia risk. Similarly, person-fit statistics (*lz* and *U3*) were associated with incident dementia (HRs = 1.22, 95% CI [1.11, 1.35] and 1.22, 95% CI [1.11, 1.36], respectively). In contrast, extreme responding was not associated with dementia risk (HR = 0.98, 95% CI [0.89, 1.07]). Pooled across all indices and studies, the overall meta-analytic effect size indicated a statistically significant and modest association between atypical response patterns and dementia risk (HR = 1.17, 95% CI [1.09, 1.27]). In models additionally adjusting for baseline cognitive test scores (Model 4), associations were further attenuated but remained significant for random response errors, multivariate outlier responses, Guttman errors, and both person-fit statistics; item-nonresponse and extreme responding were not significant.

**Figure 1:**
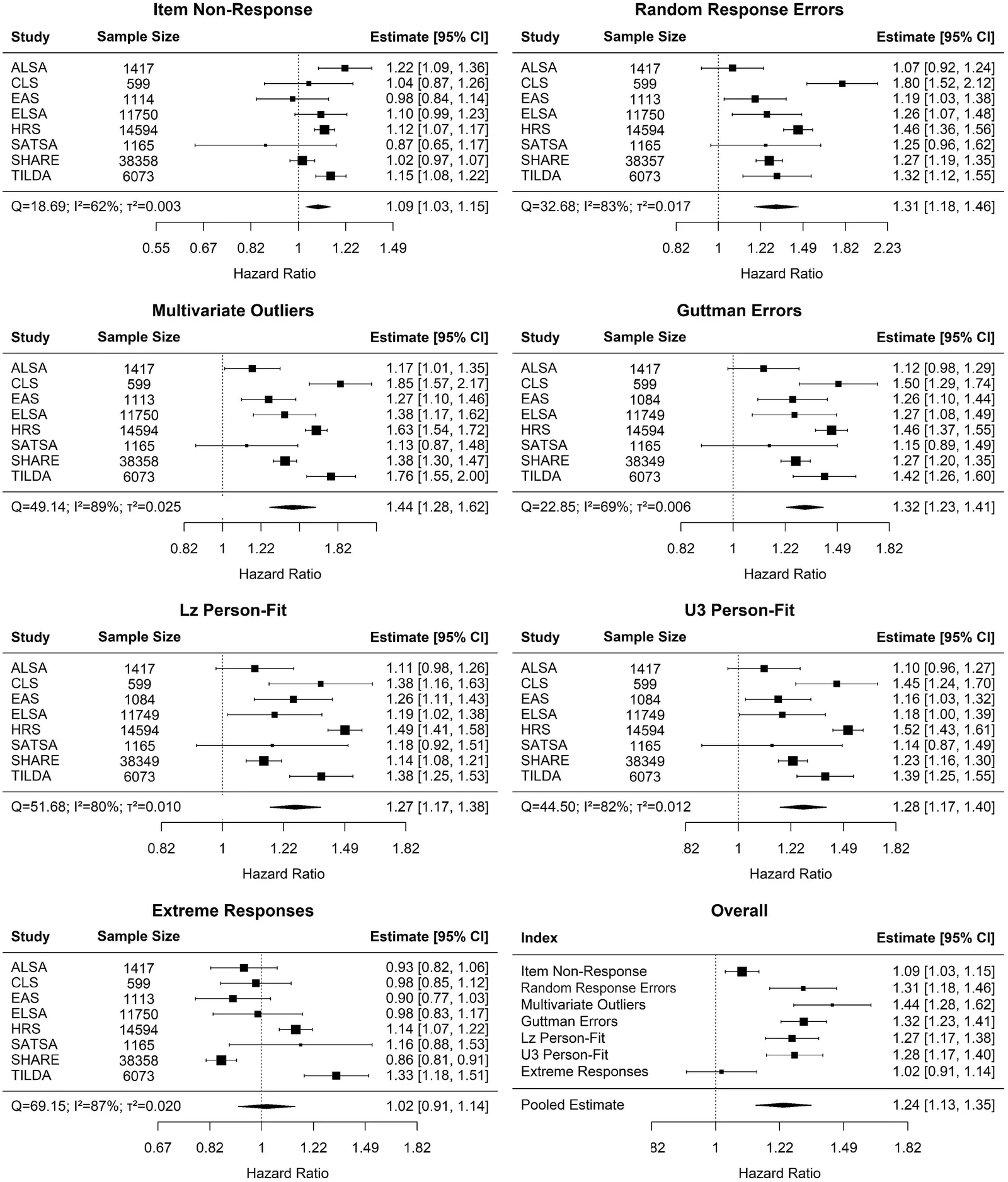
Forest plots of meta-analytic associations between survey response patterns and incident dementia across eight longitudinal aging studies. Effect sizes are adjusted for age, sex, and education in each study. τ^2^ is reported on the log-hazard-ratio scale.

**Table 2:** Meta-analytic hazard ratios for associations between survey response patterns and incident dementia.

Survey response pattern index	Model 1 (adjusted for age, sex)	Model 2 (adjusted for age, sex, education)	Model 3 (adjusted for age, sex, education, death)	Model 4 (adjusted for age, sex, education, death, baseline cognitive test)
Item non-response	1.12 (1.04, 1.19)	1.09 (1.03, 1.15)	1.06 (1.01, 1.12)	1.02 (0.99, 1.06)
Random response errors	1.37 (1.21, 1.55)	1.31 (1.18, 1.46)	1.25 (1.12, 1.40)	1.14 (1.04, 1.27)
Multivariate outlier responses	1.51 (1.30, 1.74)	1.44 (1.28, 1.62)	1.31 (1.16, 1.49)	1.18 (1.04, 1.33)
Guttman errors	1.37 (1.24, 1.52)	1.32 (1.23, 1.41)	1.25 (1.15, 1.37)	1.18 (1.13, 1.22)
*lz* person-fit statistic	1.32 (1.18, 1.49)	1.27 (1.17, 1.38)	1.22 (1.11, 1.35)	1.15 (1.08, 1.22)
*U3* person-fit statistic	1.33 (1.18, 1.50)	1.28 (1.17, 1.40)	1.22 (1.11, 1.36)	1.15 (1.07, 1.23)
Extreme responses	1.04 (0.92, 1.19)	1.02 (0.91, 1.14)	0.98 (0.89, 1.07)	0.96 (0.88, 1.04)
Overall	1.28 (1.17, 1.41)	1.24 (1.13, 1.35)	1.17 (1.09, 1.27)	1.11 (1.04, 1.17)

*Note*. Values are hazard ratios (95% confidence intervals). Models 3 and 4 account for death as a competing event using Fine and Gray’s proportional subdistribution hazards model. Model 4 additionally adjusts for individuals’ baseline cognitive test scores.

Heterogeneity in effect sizes across studies was moderate to large (*I*^2^ = 62% to 90%, τ^2^ = 0.003–0.025 across indices, see Figure 1), reflecting variability in the magnitude of associations across cohorts despite generally consistent directional effects.

### Secondary analyses: moderation by age, sex, and education

As shown in Supplementary Table 5, associations were weaker among participants ≥75 years than among younger participants (relative HRs < 1 indicating weaker associations), particularly for indicators reflecting response inconsistency and implausibility (e.g., random response errors: relative HR = 0.85, 95% CI [0.75, 0.96]; multivariate outlier responses: relative HR = 0.82, 95% CI [0.73, 0.93]). In contrast, there was little evidence that associations differed by sex, with effect sizes close to unity across all indices. Effect sizes varied by education for some indices, with stronger associations among more educated participants for indices of item non-response (secondary: relative HR = 1.11, 95% CI [1.02, 1.21]; tertiary: relative HR = 1.15, 95% CI [1.03, 1.29]) and person-fit statistics (e.g., *lz*, tertiary: relative HR = 1.18, 95% CI [1.07, 1.31]). Exploratory meta-regressions provided no clear evidence that associations differed depending on the number of survey scales contributing to the response-pattern indices (Supplementary Table 6).

## Discussion

The present study extends prior findings linking atypical survey response patterns to subsequent dementia risk in the HRS (Schneider et al., 2021) by demonstrating similar associations across eight longitudinal aging studies with substantial heterogeneity in survey content, populations, follow-up, and dementia ascertainment. Older adults who exhibited more inconsistent, implausible, or error-prone response patterns were at elevated risk of subsequently developing dementia, and associations remained evident after accounting for demographic covariates and competing mortality risk. Although associations were attenuated after adjustment for baseline cognitive test performance, several remained evident, suggesting that response-pattern indices captured information not fully reflected in standard cognitive measures. Together, these findings suggest that routine questionnaire responding contains behavioral information relevant to dementia risk.

The strongest associations were observed for indicators capturing response inconsistencies and implausible response structures, including random response errors and multivariate outlier responses, whereas extreme responding was not associated with dementia risk. This pattern suggests that the observed signal may reflect disruptions in the processes needed to understand questions, select responses, and maintain coherence across related items, rather than general response-style tendencies such as a preference for extreme response options (Schneider, 2017). For example, extreme responding reflects a consistent preference for scale endpoints, whereas random errors, Guttman errors, and low person fit reflect responses that are incompatible with related responses or the expected response structure. The latter may be more sensitive to neurocognitive difficulties in attention, working memory, or maintaining task goals, although the present study cannot distinguish these mechanisms directly.

Secondary analyses suggested that associations between response pattern indices and dementia onset were stronger among participants under age 75 and those with higher levels of education. One possible explanation is that atypical response patterns may be most informative during earlier or subtler stages of decline, when conventional indicators remain relatively insensitive, and response behaviors remain sufficiently differentiated to provide predictive information. As individuals move closer to overt dementia onset, broader cognitive and functional impairments may reduce the specificity of these behavioral signals (Gao et al., 2026). For more educated individuals, standard cognitive assessments may additionally show reduced sensitivity because of ceiling effects or cognitive reserve, whereas subtle disruptions in survey response consistency may emerge earlier (Stern, 2009, 2012).

Several limitations should be considered. Dementia ascertainment differed substantially across studies and relied on research-based rather than clinical classifications. Because cohorts used different combinations of cognitive-test cutoffs, informant/proxy reports, and self-reported diagnoses, a single harmonized definition was not feasible, and observed dementia rates should not be interpreted as directly comparable. Differential loss to follow-up, which could not be harmonized across cohorts, and variation in mortality ascertainment may also have limited identification of dementia cases before formal classification or near the end of life. Further, the mechanisms underlying these associations remain incompletely understood, as atypical response patterns may reflect multiple overlapping processes, including executive dysfunction, sensory or motor limitations, or reduced task engagement. Finally, this study focused exclusively on questionnaire response patterns, whereas modern digital surveys additionally capture rich paradata streams (e.g., response times and navigation behaviors) that may provide complementary information about cognitive functioning (Jin et al., 2023).

Overall, these findings demonstrate that atypical survey response patterns prospectively predict dementia risk across heterogeneous longitudinal studies of aging. Their primary value is for research applications rather than stand-alone clinical screening. Rather than replacing established cognitive assessment, these indicators may help researchers use behavioral information already embedded in questionnaire completion to study cognitive aging and dementia risk at scale.

## Supplementary Material

gbag197_Supplementary_Data

## Data Availability

Analytic methods and materials are available from the corresponding author upon reasonable request. This study was not preregistered.

## References

[gbag197-B1] Börsch-Supan A. , Brandt M., Hunkler C., Kneip T., Korbmacher J., Malter F., Schaan B., Stuck S., Zuber S.; SHARE Central Coordination Team. (2013). Data resource profile: The Survey of Health, Ageing and Retirement in Europe (SHARE). International Journal of Epidemiology, 42, 992–1001. 10.1093/ije/dyt088 PMC3780997 23778574

[gbag197-B2] Christensen H. , Mackinnon A., Jorm A. F., Korten A., Jacomb P., Hofer S. M., Henderson S. (2004). The Canberra Longitudinal Study: Design, aims, methodology, outcomes and recent empirical investigations. Aging Neuropsychology and Cognition, 11, 169–195. 10.1080/13825580490511053

[gbag197-B3] Clegg A. , Bandeen-Roche K., Farrin A., Forster A., Gill T. M., Gladman J., Kerse N., Lindley R., McManus R. J., Melis R., Mujica-Mota R., Raina P., Rockwood K., Teh R., van der Windt D., Witham M. (2022). New horizons in evidence-based care for older people: Individual participant data meta-analysis. Age and Ageing, 51, afac090. 10.1093/ageing/afac090 PMC9034697 35460409

[gbag197-B4] Curran P. J. , Hussong A. M. (2009). Integrative data analysis: The simultaneous analysis of multiple data sets. Psychological Methods, 14, 81–100. 10.1037/A0015914 PMC2777640 19485623

[gbag197-B5] Fine J. P. , Gray R. J. (1999). A proportional hazards model for the subdistribution of a competing risk. Journal of the American Statistical Association, 94, 496–509. 10.1080/01621459.1999.10474144

[gbag197-B6] Gao H. , Schneider S., Harris J., Hernandez R., Junghaenel D. U., Kapteyn A., Lee P. J., Stone A. A., Zelinski E., Maupin D., Orriens B., Jin H. (2026). Low-burden AI approach for cross-national early identification of cognitive impairment using real-world questionnaire response behaviours. Nature Communications, 17, 9157. 10.1038/s41467-026-76071-9 PMC13522467 42660937

[gbag197-B7] Higgins J. P. , Thompson S. G., Deeks J. J., Altman D. G. (2003). Measuring inconsistency in meta-analyses. BMJ (Clinical Research ed.), 327, 557–560. 10.1136/bmj.327.7414.557 PMC192859 12958120

[gbag197-B8] Jin H. , Junghaenel D. U., Orriens B., Lee P.-J., Schneider S. (2023). Developing early markers of cognitive decline and dementia derived from survey response behaviors: Protocol for analyses of preexisting large-scale longitudinal data. JMIR Research Protocols, 12, e44627. 10.2196/44627 PMC9993229 36809337

[gbag197-B9] Katz M. J. , Lipton R. B., Hall C. B., Zimmerman M. E., Sanders A. E., Verghese J., Dickson D. W., Derby C. A. (2012). Age-specific and sex-specific prevalence and incidence of mild cognitive impairment, dementia, and Alzheimer dementia in blacks and whites: A report from the Einstein Aging Study. Alzheimer Disease and Associated Disorders, 26, 335–343. 10.1097/WAD.0b013e31823dbcfc PMC3334445 22156756

[gbag197-B10] Knäuper B. , Carrière K., Chamandy M., Xu Z., Schwarz N., Rosen N. O. (2016). How aging affects self-reports. European Journal of Ageing, 13, 185–193. 10.1007/s10433-016-0369-0 PMC5550601 28804377

[gbag197-B11] Luszcz M. A. , Giles L. C., Anstey K. J., Browne-Yung K. C., Walker R. A., Windsor T. D. (2016). Cohort profile: The Australian longitudinal study of ageing (ALSA). International Journal of Epidemiology, 45, 1054–1063. 10.1093/ije/dyu196 PMC5841624 25468824

[gbag197-B12] Pedersen N. L. , McClearn G. E., Plomin R., Nesselroade J. R., Berg S., DeFaire U. (1991). The Swedish adoption twin study of aging: An update. Acta Geneticae Medicae et Gemellologiae, 40, 7–20. 10.1017/s0001566000006681 1950353

[gbag197-B13] Schneider S. (2017). Extracting response style bias from measures of positive and negative affect in aging research. The Journals of Gerontology, Series B: Psychological Sciences and Social Sciences, 73, 64–74. 10.1093/geronb/gbw103 PMC5926987 27543081

[gbag197-B14] Schneider S. , Hernandez R., Junghaenel D. U., Jin H., Lee P. J., Gao H., Maupin D., Orriens B., Meijer E., Stone A. A. (2024). Can you tell people’s cognitive ability level from their response patterns in questionnaires? Behavior Research Methods, 56, 6741–6758. 10.3758/s13428-024-02388-2 PMC11362444 38528247

[gbag197-B15] Schneider S. , Junghaenel D. U., Zelinski E. M., Meijer E., Stone A. A., Langa K. M., Kapteyn A. (2021). Subtle mistakes in self-report surveys predict future transition to dementia. Alzheimer’s & Dementia, 13, e12252. 10.1002/dad2.12252 PMC8652408 34934800

[gbag197-B16] Schneider S. , Lee P. J., Hernandez R., Junghaenel D. U., Stone A. A., Meijer E., Jin H., Kapteyn A., Orriens B., Zelinski E. (2024). Cognitive functioning and the quality of survey responses: An individual participant data meta-analysis of 10 epidemiological studies of aging. The Journals of Gerontology, Series B: Psychological Sciences and Social Sciences, 79, gbae030. 10.1093/geronb/gbae030 PMC10998342 38460115

[gbag197-B17] Sonnega A. , Faul J. D., Ofstedal M. B., Langa K. M., Phillips J. W., Weir D. R. (2014). Cohort profile: The health and retirement study (HRS). International Journal of Epidemiology, 43, 576–585. 10.1093/ije/dyu067 PMC3997380 24671021

[gbag197-B18] Steptoe A. , Breeze E., Banks J., Nazroo J. (2013). Cohort profile: The English longitudinal study of ageing. International Journal of Epidemiology, 42, 1640–1648. 10.1093/ije/dys168 PMC3900867 23143611

[gbag197-B19] Stern Y. (2009). Cognitive reserve. Neuropsychologia, 47, 2015–2028. 10.1016/j.neuropsychologia.2009.03.004 PMC2739591 19467352

[gbag197-B20] Stern Y. (2012). Cognitive reserve in ageing and Alzheimer’s disease. The Lancet Neurology, 11, 1006–1012. 10.1016/S1474-4422(12)70191-6(12)70191-6 PMC3507991 23079557

[gbag197-B21] Tourangeau R. , Rips L. J., Rasinski K. (2000). The Psychology of Survey Response. Cambridge University Press. 10.2307/3085796

[gbag197-B22] Verlinden V. J. , van der Geest J. N., de Bruijn R. F., Hofman A., Koudstaal P. J., Ikram M. A. (2016). Trajectories of decline in cognition and daily functioning in preclinical dementia. Alzheimer’s & Dementia, 12, 144–153. 10.1016/j.jalz.2015.08.001 26362597

[gbag197-B23] Viechtbauer W. (2010). Conducting meta-analyses in R with the metafor package. Journal of Statistical Software, 36, 1–48. 10.18637/jss.v036.i03

[gbag197-B24] Whelan B. J. , Savva G. M. (2013). Design and methodology of the Irish Longitudinal Study on Ageing. Journal of the American Geriatrics Society, 61 Suppl 2, S265–S268. 10.1111/jgs.12199 23662718

