# Supplementary material for: Atypical survey response patterns as early indicators of dementia onset: a coordinated analysis across longitudinal aging cohorts
Source: J Gerontol B Psychol Sci Soc Sci. 2026 Sep 18;81(12):gbag197. doi: 10.1093/geronb/gbag197 (PMC13760214; doi:10.1093/geronb/gbag197)
Supplement: Supplementary file 1 [file gbag197_supplementary_data.docx]

***The Journals of Gerontology, Series B: Psychological Sciences and Social Sciences* Supplementary Material: Schneider et al. Atypical survey response patterns as early indicators of dementia onset: A coordinated analysis across longitudinal aging cohorts.**

Table of Contents

[Supplementary Table 1.1-1.8. Description of included studies and questionnaires	2](#_Toc235802598)

[1.1. Australian Longitudinal Ageing Study (ALSA)	2](#_Toc235802599)

[1.2. Canberra Longitudinal Study (CLS)	3](#_Toc235802600)

[1.3. Einstein Aging Study (EAS)	4](#_Toc235802601)

[1.4. English Longitudinal Study of Ageing (ELSA)	5](#_Toc235802602)

[1.5. Health and Retirement Study (HRS)	6](#_Toc235802603)

[1.6. Swedish Adoption Twin Study of Aging (SATSA)	7](#_Toc235802604)

[1.7. Survey of Health, Ageing and Retirement in Europe (SHARE)	8](#_Toc235802605)

[1.8. The Irish Longitudinal Study on Ageing (TILDA)	9](#_Toc235802606)

[Supplementary Table 2. Description of survey response pattern indicators	10](#_Toc235802607)

[Supplementary Table 3. Baseline cognitive measures used as covariates in Model 4.	12](#_Toc235802608)

[Supplementary Table 4. Proportional-hazards assumption checks using covariate-by-log-time interactions.	13](#_Toc235802609)

[Supplementary Table 5. Demographic moderation of response-pattern associations with incident dementia	15](#_Toc235802610)

[Supplementary Table 6. Study-level meta-regression testing whether response-pattern associations varied by number of survey scales	16](#_Toc235802611)

[References	17](#_Toc235802612)

# Supplementary Table 1.1-1.8. Description of included studies and questionnaires

| 1.1. Australian Longitudinal Ageing Study (ALSA) | | | |
| --- | --- | --- | --- |
| A panel survey exploring factors associated with age-related changes in health and well-being of persons aged 56 years and older in South Australia. It was initiated in 1992 and has surveyed participants every 2 years for 13 waves. Data for 10 waves (except waves 2, 4, and 5 as they comprised only shorter household interviews, no clinical assessment nor self-administered questionnaires) collected between 1992 and 2014 were included in the present study. Survey response pattern indices were exclusively derived from wave 1 (baseline wave), and subsequent waves were used to determine dementia status. | | | |
|  | **Baseline Wave** | | |
|  |  |  | **W1** |
| Participants starting at wave |  |  | 1420 |
| **Questionnaires** | **# items** | **# resp. options** | **Cronbach's Alpha** |
| General Locus of Control Scale | 12 | 5 | .72 |
| Health Locus of Control - Internal | 6 | 5 | .73 |
| Health Locus of Control - Chance | 6 | 5 | .65 |
| Health Locus of Control - Powerful others | 6 | 5 | .70 |
| Metamemory in Adulthood - Task | 16 | 5 | .74 |
| Metamemory in Adulthood - Locus | 9 | 5 | .71 |
| Metamemory in Adulthood - Capacity | 17 | 5 | .86 |
| Oral Health Impact Profile | 10 | 5 | .80 |
| Self-Esteem Scale | 5 | 5 | .80 |
| Nagi Mobility Scale | 5 | 4 | .79 |
| CES-D subscale - Depressed affect/mood | 5 | 4 | .64 |
| CES-D subscale - Somatic and retarded activity | 4 | 4 | .61 |
| CES-D subscale - Positive affect/well-being | 7 | 4 | .77 |
| Adelaide Activities Profile - Domestic chores | 6 | 4 | .59 |
| Adelaide Activities Profile - Household maintenance | 8 | 5 | .80 |

| 1.2. Canberra Longitudinal Study (CLS) | | | |
| --- | --- | --- | --- |
| An epidemiological study of mental health and cognitive functioning that surveyed community-based adults aged 70 years and older residing in the Canberra area in Australia. Participants were interviewed at 4 waves in 4-year intervals from 1990-2002. Survey response pattern indices were exclusively derived from wave 1 (baseline wave), and subsequent waves were used to determine dementia status. | | | |
|  | **Baseline Wave** | | |
| **Baseline Wave** |  |  | **W1** |
| Participants starting at wave |  |  | 600 |
| **Questionnaires** | **# items** | **# resp. options** | **Cronbach's Alpha** |
| Eye Sight (can you see to read) | 4 | 3 | .92 |
| Hearing | 4 | 3 | .85 |
| Social Participation | 4 | 5/6 | .66 |
| ADL | 7 | 3/4/5 | .86 |
| IADL | 6 | 3/4 | .85 |
| Subjective Cognitive Decline | 6 | 3 | .73 |
| Contact with Social Network | 4 | 5 | .58 |
| Positive Feelings on Life and Aging | 12 | 5 | .80 |
| Negative Feelings on Life and Aging | 8 | 5 | .74 |
| Internal Locus of Control | 6 | 6 | .62 |
| External Locus of Control | 8 | 6 | .74 |
| Change in Social Participation compared to 5 years ago | 5 | 3 | .59 |

| 1.3. Einstein Aging Study (EAS) | | | | | | | | |
| --- | --- | --- | --- | --- | --- | --- | --- | --- |
| Recruited community-dwelling, English-speaking, aged 70 years and older Bronx, N.Y. residents from Health Care Financing Administration, Centers for Medicaid and Medicare Services rosters for Medicare-eligible persons (1993-2004), then from New York City Board of Elections (2004 onwards). Annual interview data collected from 1993 to 2017 were included in the present study. Survey response pattern indices were derived from each participant’s personal baseline wave, and subsequent waves were used to determine dementia status. | | | | | | | | |
|  | | | **Baseline Wave** | | | | | |
|  |  |  | **W7** | **W8** | **W9** | **W10** | **W11** | **W12** |
| Participants starting at wave |  |  | 142 | 156 | 174 | 42 | 1 | 1 |
| **Questionnaires** | **# items** | **# resp. options** | **Cronbach's Alpha^a^** | | | | | |
| ADL physical self-maintenance | 6 | 3/5 | .49 | .68 | .58 | .85 | .69 | .81 |
| IADL functional status | 8 | 3/4/5 | .76 | .77 | .72 | .83 | .83 | .83 |
| SF-36 Physical functioning | 10 | 3 | .88 | .87 | .89 | .89 | .90 | .90 |
| SF-36 Energy/fatigue | 4 | 6 | .84 | .83 | .81 | .82 | .81 | .79 |
| SF-36 Emotional well-being | 5 | 6 | .81 | .84 | .84 | .80 | .82 | .83 |
| SF-36 General health | 5 | 5 | .71 | .69 | .74 | .70 | .68 | .63 |

**Einstein Aging Study (EAS), continued**

|  |  |  | **W13** | **W14** | **W15** | **W16** | **W17** | **W18** |
| --- | --- | --- | --- | --- | --- | --- | --- | --- |
| Participants starting at wave |  |  | 86 | 144 | 102 | 25 | 48 | 22 |
| **Questionnaires** | **# items** | **# resp. options** | **Cronbach's Alpha^a^** | | | | | |
| ADL physical self-maintenance | 6 | 3/5 | .37 | .90 | .74 | .76 | .56 | .43 |
| IADL functional status | 8 | 3/4/5 | .77 | .78 | .74 | .82 | .79 | .80 |
| SF-36 Physical functioning | 10 | 3 | .90 | .89 | .88 | .88 | .88 | .89 |
| SF-36 Energy/fatigue | 4 | 6 | .81 | .81 | .82 | .85 | .85 | .85 |
| SF-36 Emotional well-being | 5 | 6 | .85 | .78 | .81 | .84 | .87 | .84 |
| SF-36 General health | 5 | 5 | .69 | .65 | .67 | .69 | .70 | .69 |

**Einstein Aging Study (EAS), continued**

|  |  |  | **W19** | **W20** | **W21** | **W22** | **W23** |
| --- | --- | --- | --- | --- | --- | --- | --- |
| Participants starting at wave |  |  | 2 | 57 | 42 | 59 | 11 |
| **Questionnaires** | **# items** | **# resp. options** | **Cronbach's Alpha^a^** | | | | |
| ADL physical self-maintenance | 6 | 3/5 | .62 | .29 | .47 | .84 | .29 |
| IADL functional status | 8 | 3/4/5 | .84 | .81 | .77 | .80 | .64 |
| SF-36 Physical functioning | 10 | 3 | .89 | .89 | .89 | .89 | .89 |
| SF-36 Energy/fatigue | 4 | 6 | .81 | .79 | .84 | .83 | .81 |
| SF-36 Emotional well-being | 5 | 6 | .84 | .86 | .81 | .81 | .81 |
| SF-36 General health | 5 | 5 | .67 | .65 | .64 | .66 | .69 |

^a^ Cronbach’s alpha coefficients were calculated using all respondents from the original survey wave, not only participants included in the present analytic sample.

| 1.4. English Longitudinal Study of Ageing (ELSA) | | | | | | | | | | | | |
| --- | --- | --- | --- | --- | --- | --- | --- | --- | --- | --- | --- | --- |
| Composed primarily of respondents aged 50 years and older living in England, who participated in the Health Survey for England (HSE) in 1998, 1999, and 2001. It was initiated in 2002 and has surveyed participants every 2 years for 9 waves (2002-2018). Survey response pattern indices were derived from each participant’s personal baseline wave; subsequent waves were used to determine dementia status. | | | | | | | | | | | | |
|  |  | | | **Baseline Wave** | | | | | | | |  |
|  | |  |  | **W1** | **W2** | **W3** | **W4** | **W5** | **W6** | **W7** | **W8** | |
| Participants starting at wave | |  |  | 8097 | 441 | 969 | 2012 | 289 | 728 | 322 | 55 | |
| **Questionnaires** | | **# items** | **# resp. options** | **Cronbach’s Alpha^a^** | | | | | | | | |
| CASP-19 Control | | 6 | 4 | .63 | .64 | .69 | .68 | .69 | .70 | .67 | .68 | |
| CASP-19 Autonomy | | 5 | 4 | .52 | .54 | .56 | .56 | .58 | .57 | .57 | .57 | |
| CASP-19 Pleasure | | 4 | 4 | .65 | .68 | .67 | .68 | .68 | .70 | .70 | .69 | |
| CASP-19 Self-realization | | 4 | 4 | .79 | .80 | .79 | .80 | .80 | .80 | .80 | .80 | |
| Cultural participation | | 4 | 6 | .70 | .77 | .76 | .77 | .77 | .77 | .77 | .77 | |
| Accessing local amenities | | 5 | 5 (4 at W1W2) | .94 | .93 |  | .90 | .91 | .92 |  |  | |
| Accessing healthcare amenities | | 5 | 5 (4 at W1W2) | .92 | .91 |  | .91 | .91 | .92 |  |  | |
| GHQ-12 Social dysfunction | | 6 | 4 | .80 |  | .77 |  |  |  |  |  | |
| GHQ-12 General dysphoria | | 6 | 4 | .86 |  | .87 |  |  |  |  |  | |
| Control and demand | | 5 | 6 | .53 | .51 | .50 | .51 | .56 | .57 |  |  | |
| Neighborhood disorder | | 3 | 7 | .61 |  | .60 |  |  |  | .59 |  | |
| Neighborhood cohesion | | 6 | 7 | .74 |  | .77 |  |  |  | .77 |  | |
| Loneliness | | 5 (4 at W2) | 3 |  | .69 | .78 | .77 | .77 | .77 | .75 | .76 | |
| Diener/Satisfaction with life | | 5 | 7 |  | .89 | .90 | .89 | .90 | .90 | .90 | .90 | |
| Positive attitude on aging | | 6 | 5 |  | .50 |  |  |  |  | .50 | .50 | |
| Negative attitude on aging | | 6 | 5 |  | .72 |  |  |  |  | .71 | .72 | |
| Altruistic tendency | | 5 | 5 |  |  |  | .77 |  |  |  |  | |
| Concern about ingroup relationships - family | | 4 | 6 |  |  |  | .80 |  |  |  |  | |
| Concern about ingroup relationships - friends | | 5 | 6 |  |  |  | .88 |  |  |  |  | |
| Religious feelings and behavior | | 4 | 4 |  |  |  |  | .92 |  |  |  | |
| Positive affect | | 13 | 5 |  |  |  |  | .94 |  |  |  | |
| Big5 Extraversion | | 5 | 4 |  |  |  |  | .75 |  |  |  | |
| Big5 Agreeableness | | 5 | 4 |  |  |  |  | .80 |  |  |  | |
| Big5 Neuroticism | | 4 | 4 |  |  |  |  | .68 |  |  |  | |
| Big5 Conscientiousness | | 5 | 4 |  |  |  |  | .67 |  |  |  | |
| Big5 Openness | | 7 | 4 |  |  |  |  | .79 |  |  |  | |
| Perceived discrimination | | 5 | 6 |  |  |  |  | .78 |  |  |  | |

^a^ Cronbach’s alpha coefficients were calculated using all respondents from the original survey wave, not only participants included in the present analytic sample.

| 1.5. Health and Retirement Study (HRS) | | | | | | | | |
| --- | --- | --- | --- | --- | --- | --- | --- | --- |
| The study recruited a nationally representative sample of the population aged 50 years and older, living in private households in the United States, initiated in 1992. Psychosocial leave-behind survey provided to each half-sample of respondents (randomly selected) every two waves starting at Wave 8. This research included half-samples alternate waves up to Wave 15 (2006-2020). Survey response pattern indices were derived from each participant’s personal baseline wave, and subsequent waves were used to determine dementia status. | | | | | | | | |
|  | | | **Baseline Wave** | | | | | |
|  |  |  | **W8** | **W9** | **W10** | **W11** | **W12** | **W13** |
| Participants starting at wave |  |  | 5613 | 4796 | 1764 | 1497 | 306 | 620 |
| **Questionnaires** | **# items** | **# resp. options** | **Cronbach’s Alpha^a^** | | | | | |
| Diener/Satisfaction with life | 5 | 7 (6 at W8) | .88 | .87 | .89 | .88 | .89 | .88 |
| Loneliness | 3 | 3 | .82 | .80 | .81 | .80 | .81 | .80 |
| Neighborhood disorder | 4 | 7 | .64 | .83 | .83 | .83 | .84 | .85 |
| Neighborhood cohesion | 4 | 7 | .82 | .86 | .86 | .86 | .86 | .87 |
| Perceived constraints on personal control | 5 | 6 | .86 | .86 | .86 | .86 | .86 | .86 |
| Perceived mastery | 5 | 6 | .89 | .89 | .90 | .90 | .90 | .90 |
| Religiosity | 4 | 6 | .92 | .92 | .93 | .92 | .93 | .93 |
| Perceived discrimination | 5 | 6 | .79 | .80 | .79 | .81 | .81 | .80 |
| Big5 Extraversion | 5 | 4 | .75 | .73 | .75 | .75 | .76 | .74 |
| Big5 Agreeableness | 5 | 4 | .78 | .77 | .78 | .78 | .79 | .78 |
| Big5 Neuroticism | 4 | 4 | .71 | .72 | .71 | .72 | .71 | .70 |
| Big5 Conscientiousness | 5 | 4 | .65 | .66 | .66 | .67 | .66 | .66 |
| Big5 Openness | 7 | 4 | .78 | .79 | .79 | .79 | .80 | .80 |
| Purpose in life | 7 | 6 | .73 | .75 | .77 | .77 | .76 | .76 |
| Hostility | 5 | 6 | .79 | .78 | .79 | .78 |  |  |
| LOT-R Optimism | 6 | 6 | .73 | .74 | .75 | .74 |  |  |
| Hopelessness | 4 | 6 | .86 | .84 | .85 | .87 |  |  |
| Social effort/reward balance | 3 | 5 | .78 | .77 | .77 | .76 |  |  |
| Anxiety | 5 | 4 | .81 | .82 | .82 | .82 |  |  |
| Anger in | 4 | 4 | .78 | .78 | .79 | .81 |  |  |
| Anger out | 7 | 4 | .87 | .87 | .87 | .89 |  |  |

^a^ Cronbach’s alpha coefficients were calculated using all respondents from the original survey wave, not only participants included in the present analytic sample.

| 1.6. Swedish Adoption Twin Study of Aging (SATSA) | | | | | | | |
| --- | --- | --- | --- | --- | --- | --- | --- |
| The study was designed to examine genetic and environmental influences contributing to individual differences in aging. It was started in 1984 with a sample of Swedish twins 26 years and older who were raised together or apart from one another. We included participants 50 years and older who self-completed mail-out questionnaires at 6 waves that were conducted in 3-year intervals between 1984 and 1993 and additionally in 2004 and 2007. Survey response pattern indices were derived from each participant’s personal baseline wave, and subsequent waves were used to determine dementia status. | | | | | | | |
|  | | | **Baseline Wave** | | | | |
|  |  |  | | **W1** | **W2** | **W3** | **W4** |
| Participants starting at wave |  |  | | 1117 | 96 | 37 | 7 |
| **Questionnaires** | **# items** | **# resp. options** | | **Cronbach's Alpha^a^** | | | |
| EAS scale - emotionality | 4 | 5 | | .31 | .27 | .27 | .32 |
| EAS scale - fear | 4 | 5 | | .61 | .60 | .56 | .62 |
| EAS scale - anger | 4 | 5 | | .52 | .49 | .49 | .54 |
| EAS scale - active | 4 | 5 | | .73 | .73 | .73 | .75 |
| EAS scale - sociable | 4 | 5 | | .55 | .52 | .57 | .57 |
| KSP scale - impulsive | 10 | 5 | | .71 | .67 | .69 | .72 |
| KSP scale - monotony avoidance, sensation seeker | 10 | 5 | | .74 | .73 | .73 | .76 |
| Life satisfaction | 13 | 5 | | .79 | .80 | .79 | .80 |
| Locus of control scale - Luck | 4 | 5 | | .30 | .30 | .25 | .30 |
| Locus of control scale - Responsibility | 4 | 5 | | .64 | .62 | .64 | .66 |
| Locus of control scale - Life direction | 4 | 5 | | .47 | .49 | .44 | .55 |
| Hostility subscale - Paranoid alienation | 5 | 5 | | .63 | .64 | .65 | .67 |
| Hostility subscale - Cynicism | 5 | 5 | | .73 | .72 | .74 | .74 |
| Lack of assertiveness (Inhibition of aggression) | 10 | 5 | | .68 | .66 | .67 | .71 |
| ADL | 7 | 3 | | .79 | .76 | .68 | .67 |
| IADL | 7 | 3 | | .86 |  |  | .89 |
| State anxiety - Positive items | 4 | 5 | | .87 | .85 | .87 | .85 |
| State anxiety - Negative items | 6 | 5 | | .89 | .90 | .90 | .91 |
| Type A behavior subscale – Hard-driving | 5 | 5 | | .53 | .49 | .45 | .48 |
| Type A behavior subscale - Stress | 7 | 5 | | .74 |  |  |  |
| Type A behavior subscale - Ambtiousness | 4 | 5 | | .56 |  |  |  |
| NEO personality scale - Openness to experience | 6 | 5 | | .64 | .63 | .70 | .71 |
| NEO personality scale - Agreeableness | 10 | 5 | | .48 |  |  |  |
| NEO personality scale - Conscientiousness | 10 | 5 | | .65 |  |  |  |
| CES-D subscale - Depressed mood | 6 | 4 | |  | .87 | .83 | .84 |
| CES-D subscale - Psychomotor retardation | 6 | 4 | |  | .79 | .76 | .79 |
| CES-D subscale - Lack of well-being | 4 | 4 | |  | .72 | .66 | .68 |
| Optimism scale (positive items) | 4 | 5 | |  | .63 | .67 | .67 |
| Pessimism scale (negative items) | 4 | 5 | |  | .68 | .70 | .78 |
| Other physical ADL | 7 | 3 | |  | .89 | .87 | .88 |
| Activities | 7 | 5 | |  |  |  | .54 |

^a^ Cronbach’s alpha coefficients were calculated using all respondents from the original survey wave, not only participants included in the present analytic sample.

| 1.7. Survey of Health, Ageing and Retirement in Europe (SHARE) | | | | | | | | | |
| --- | --- | --- | --- | --- | --- | --- | --- | --- | --- |
| The study has surveyed participants aged 50 years and older in 2-year intervals for 8 waves from 2004 to 2020 to study the effects of socio-economic and environmental policies in European countries and Israel. We included interviewer-administered core interviews and the drop-off surveys (not administered in waves 3 and 8). Survey response pattern indices were derived from each participant’s personal baseline wave, and subsequent waves were used to determine dementia status. | | | | | | | | | |
|  | | | | **Baseline Wave** | | | | | |
|  |  |  | **W1** | | **W2** | **W4** | **W5** | **W6** | **W7** |
| Participants starting at wave |  |  | 10112 | | 4680 | 13788 | 6783 | 2831 | 182 |
| **Questionnaires** | **# items** | **# resp. options** | **Cronbach's Alpha^a^** | | | | | | |
| CASP-12: Control | 3 | 4 | .70 | | .72 | .69 | .72 | .72 | .69 |
| CASP-12: Autonomy | 3 | 4 | .40 | | .33 | .32 | .25 | .36 | .31 |
| CASP-12: Pleasure | 3 | 4 | .68 | | .65 | .64 | .67 | .65 | .70 |
| CASP-12: Self-realization | 3 | 4 | .81 | | .82 | .82 | .81 | .81 | .82 |
| LOT-R, Optimism - Pessimism | 7 | 5 | .74 | | .73 |  |  |  |  |
| Family duties | 4 | 5 | .79 | | .78 |  |  |  |  |
| Family responsibility vs State | 3 | 5 | .80 | | .78 |  |  |  |  |
| CES-D: Depression/Feelings | 5 | 4 | .79 | |  |  |  |  |  |
| CES-D: Somatic complaints | 7 | 4 | .79 | |  |  |  |  |  |
| Expectations of others | 4 | 5 | .61 | |  |  |  |  |  |
| Health care advice from your usual doctor or nurse | 5 | 3 |  | | .77 |  |  |  |  |
| Health - Talk about problems | 4 | 4 |  | | .89 |  |  |  |  |
| Health - Explanations/listening | 3 | 4 |  | | .88 |  |  |  |  |
| Loneliness | 4 | 3 |  | |  | .84 | .80 | .81 |  |
| Anxiety | 5 | 4 |  | |  | .71 | .72 |  |  |
| Self-rated health state | 5 | 3 |  | |  | .78 |  |  |  |

^a^ Cronbach’s alpha coefficients were calculated using all respondents from the original survey wave, not only participants included in the present analytic sample.

|  | 1.8. The Irish Longitudinal Study on Ageing (TILDA) | | | | | |
| --- | --- | --- | --- | --- | --- | --- |
|  | The study has been surveying residents of Ireland aged 50 years and older since 2010. Self-administered questionnaires were completed in 2-year intervals for 4 waves between 2010 and 2016. Survey response pattern indices were derived from each participant’s personal baseline wave, and subsequent waves were used to determine dementia status. | | | | | |
|  | | | | **Baseline Wave** | | |
|  |  |  |  | **W1** | **W2** | **W3** |
|  | Participants starting at wave |  |  | 5791 | 229 | 54 |
|  | **Questionnaires** | **# items** | **# resp. options** | **Cronbach's Alpha^a^** | | |
|  | Social activities participation - active or social leisure | 7 | 8 | .59 | .58 | .57 |
|  | Social activities participation - passive or solitary leisure | 5 (4 at W3) | 8 | .45 | .49 | .52 |
|  | Loneliness | 5 | 3 | .79 | .80 | .80 |
|  | PSS-4 | 4 | 5 | .62 | .58 | .67 |
|  | Friendship | 7 | 4 | .72 | .72 | .72 |
|  | Penn State Worry scale | 8 | 5 | .94 | .94 | .94 |
|  | CASP-19 Control | 4 | 4 | .59 |  | .61 |
|  | CASP-19 Autonomy | 5 (3 at W3) | 4 | .52 |  | .30 |
|  | CASP-19 Pleasure | 5 (3 at W3) | 4 | .78 |  | .73 |
|  | CASP-19 Self-realization | 5 | 4 | .80 |  |  |
|  | Accommodation problems | 19 | 4 |  | .93 | .93 |
|  | Concerns about falling | 16 | 4 |  | .95 | .95 |
|  | Neighborhood disorder | 3 | 7 |  | .52 |  |
|  | Neighborhood cohesion | 6 | 7 |  | .70 |  |
|  | Anxiety | 7 | 4 | .84 |  |  |
|  | APQ-32 Timeline chronic | 5 | 5 | .85 |  |  |
|  | APQ-32 Timeline cyclical | 5 | 5 | .87 |  |  |
|  | APQ-32 Emotional representations | 5 | 5 | .86 |  |  |
|  | APQ-32 Control positive | 5 | 5 | .84 |  |  |
|  | APQ-32 Control negative | 4 | 5 | .81 |  |  |
|  | APQ-32 Consequences positive | 3 | 5 | .78 |  |  |
|  | APQ-32 Consequences negative | 5 | 5 | .88 |  |  |

^a^ Cronbach’s alpha coefficients were calculated using all respondents from the original survey wave, not only participants included in the present analytic sample.

# Supplementary Table 2. Description of survey response pattern indicators

| **Indicator** | **Rationale** | **Computation** |
| --- | --- | --- |
| Item non-response | Skipping survey questions may reflect difficulties with comprehension, memory retrieval, decision-making, or sustained task engagement. Increased item nonresponse has been associated with lower cognitive functioning in older adults. | We calculated the proportion of missing values per respondent and scale in each study using SAS version 9.4 (Cary, NJ). The scores were arcsine transformed to stabilize the variance of the calculated proportions. |
| Random response errors | Inconsistent responses within a questionnaire may reflect reduced attentional control, uncertainty, or difficulty maintaining stable response mappings across items. Greater random response variability may therefore capture subtle disruptions in cognitive efficiency or self-monitoring. | To derive an index of random measurement error around each person’s “true” scale scores, we first transformed all survey items onto a common 0-10 metric. We then applied a single-factor model to each scale, saved the residual errors (i.e., the difference between a person’s observed and predicted scores for each item), and computed the variance of the item residuals for each person. The factor models were estimated using M*plus* version 8.9 (Muthén & Muthén, 2017). Missing values were accommodated with full information maximum likelihood estimation. The residual error variances were square root transformed to normalize their distribution. |
| Multivariate outlier responses | Highly atypical response patterns may reflect difficulties integrating questionnaire content or maintaining coherent response strategies across items. Such globally unusual response profiles may indicate cognitive or attentional disruption. | We computed the squared Mahalanobis distance of each respondent’s response pattern per scale using M*plus.* Missing values were accommodated with maximum likelihood estimation. To place the Mahalanobis distances on a common (0 to 1) metric for each scale, they were transformed using the chi-square cumulative distribution function. |
| Guttman errors | Violations of expected item response ordering may reflect difficulties processing item structure consistently or maintaining internally coherent response patterns. Increased Guttman errors may therefore indicate subtle impairments in cognitive control or comprehension. | We used the R package *PerFit* (Tendeiro, Meijer, & Niessen, 2016) to obtain the number of Guttman errors for ordered categorical (i.e., polytomous) responses per scale in each study. The program accommodates missing values via Hotdeck imputation. The Guttman errors were normed to a 0-1 range using the recursive algorithm by Emons (2008) and log-transformed (a constant of 0.1 was added to allow log-transformation of zero values). |
| *lz* person-fit statistic | Parametric person-fit statistics provide a model-based assessment of whether an individual’s response pattern is consistent with expectations under an item response theory (IRT) model. The *lz* statistic quantifies the extent to which a respondent’s pattern deviates from the most likely pattern given their estimated trait level. | Computed using the R package *PerFit* for polytomous responses per questionnaire. Missing values were handled via hot-deck imputation. Scores were normalized to a 0–1 range and log-transformed using the same procedure applied to Guttman errors. |
| U3 person-fit statistic | The U3 statistic quantifies aberrant response patterns that violate expected item ordering. Higher U3 values may indicate difficulties maintaining consistent response strategies across related items, offering a complementary perspective to model-based fit indices. | Computed using the R package *PerFit* for polytomous responses per questionnaire. Missing values were handled via hot-deck imputation. Scores were normalized to a 0–1 range and log-transformed using the same procedure applied to Guttman errors. |
| Extreme responses | Excessive selection of extreme response options may reflect difficulties using response scales in a differentiated manner or reliance on simplified response strategies during survey completion. | Extreme responding was calculated per questionnaire using a two-dimensional nominal response model (NRM). The NRM addresses limitations of methods that merely count the number of times that a participant selects an extreme response. Whereas these methods confound the measurement of response behaviors and a participant’s extremeness on the latent trait measured, the NRM separates these based aspects by extracting them as two separate latent factors from the response pattern. The NRM was estimated in M*plus* using maximum likelihood estimation (Falk & Ju, 2020; Schneider, 2018). |

# Supplementary Table 3. Baseline cognitive measures used as covariates in Model 4.

| **Study** | **Cognitive measures** |
| --- | --- |
| ALSA | Mini-mental state exam (MMSE) |
| CLS | Mini-mental state exam (MMSE) |
| EAS | Blessed Information-Memory-Concentration (BIMC) test |
| ELSA | Immediate recall, delayed recall, orientation |
| HRS | Modified Telephone Interview for Cognitive Status (TICS) |
| SATSA | Mini-mental state exam (MMSE) |
| SHARE | Immediate recall, delayed recall |

*Note*. Cognitive measures were assessed at the same baseline wave used to derive the response-pattern indices. TILDA is not shown because Model 4 builds on Model 3, which did not include TILDA because mortality data were unavailable.

# Supplementary Table 4. Proportional-hazards assumption checks using covariate-by-log-time interactions.

| **Study** | **Time-interaction terms** | **Estimate** | **SE** | **Raw P-value** | **Corrected P-value** |
| --- | --- | --- | --- | --- | --- |
| ALSA | log(time)*index | .013 | .086 | .876 | 1.00 |
|  | log(time)*sex | -.059 | .149 | .693 | 1.00 |
|  | log(time)*age | -.010 | .014 | .459 | 1.00 |
|  | log(time)*education | .143 | .163 | .383 | 1.00 |
| CLS | log(time)*index | -.424 | .184 | .021 | .168 |
|  | log(time)*sex | .145 | .285 | .611 | 1.00 |
|  | log(time)*age | .037 | .033 | .262 | 1.00 |
|  | log(time)*edu_secondary | -.532 | .811 | .512 | 1.00 |
|  | log(time)*edu_tertiary | -.233 | .825 | .778 | 1.00 |
| EAS | log(time)*index | -.143 | .099 | .152 | 1.00 |
|  | log(time)*sex | .116 | .189 | .538 | 1.00 |
|  | log(time)*age | .031 | .019 | .114 | .913 |
|  | log(time)*edu_secondary | .279 | .314 | .374 | 1.00 |
|  | log(time)*edu_tertiary | .423 | .314 | .178 | 1.00 |
| ELSA | log(time)*index | -.012 | .137 | .929 | 1.00 |
|  | log(time)*sex | -.649 | .265 | .014 | .114 |
|  | log(time)*age | .016 | .015 | .294 | 1.00 |
|  | log(time)*edu_secondary | .181 | .347 | .601 | 1.00 |
|  | log(time)*edu_tertiary | .639 | .342 | .062 | .494 |
| HRS | log(time)*index | .028 | .068 | .679 | 1.00 |
|  | log(time)*sex | .105 | .146 | .473 | 1.00 |
|  | log(time)*age | .022 | .010 | .023 | .182 |
|  | log(time)*edu_secondary | -.023 | .170 | .891 | 1.00 |
|  | log(time)*edu_tertiary | .390 | .199 | .051 | .404 |
| SATSA | log(time)*index | -.016 | .229 | .946 | 1.00 |
|  | log(time)*sex | -.389 | .500 | .437 | 1.00 |
|  | log(time)*age | .046 | .029 | .113 | .900 |
|  | log(time)*edu_secondary | .924 | .632 | .144 | 1.00 |
|  | log(time)*edu_tertiary | 1.110 | .831 | .181 | 1.00 |
| SHARE | log(time)*index | -.114 | .052 | .030 | .238 |
|  | log(time)*sex | -.116 | .104 | .265 | 1.00 |
|  | log(time)*age | .003 | .006 | .570 | 1.00 |
|  | log(time)*edu_secondary | -.109 | .116 | .348 | 1.00 |
|  | log(time)*edu_tertiary | -.223 | .160 | .163 | 1.00 |
| TILDA | log(time)*index | .085 | .134 | .523 | 1.00 |
|  | log(time)*sex | -.088 | .272 | .745 | 1.00 |
|  | log(time)*age | .010 | .018 | .596 | 1.00 |
|  | log(time)*edu_secondary | .213 | .316 | .500 | 1.00 |
|  | log(time)*edu_tertiary | .375 | .640 | .557 | 1.00 |

*Note.* Time-interaction terms test whether covariate effects vary over follow-up time. Adjusted p values are Bonferroni-corrected within study for the number of time-interaction tests. Checks were conducted in representative adjusted Cox models using Guttman errors as the response-pattern index.

# Supplementary Table 5. Demographic moderation of response-pattern associations with incident dementia

| **Survey response pattern indicator** | **Age ≥75 years (vs. <75 years)** | **Male (vs. female)** | **Secondary education (vs. primary)** | **Tertiary education (vs. primary)** |
| --- | --- | --- | --- | --- |
| Item non-response | 0.96  (0.86, 1.07) | 1.01  (0.91, 1.12) | 1.11  (1.02, 1.21) | 1.15  (1.03, 1.29) |
| Random response errors | 0.85  (0.75, 0.96) | 1.08  (0.95, 1.22) | 1.04  (0.93, 1.16) | 1.07  (0.96, 1.21) |
| Multivariate outlier responses | 0.82  (0.73, 0.93) | 1.07  (0.95, 1.21) | 1.08  (0.98, 1.19) | 1.07  (0.96, 1.19) |
| Guttman errors | 0.87  (0.78, 0.98) | 1.09  (0.97, 1.22) | 1.09  (0.99, 1.20) | 1.10  (0.97, 1.25) |
| *lz* person-fit statistic | 0.90  (0.80, 1.02) | 1.07  (0.95, 1.21) | 1.10  (1.01, 1.19) | 1.18  (1.07, 1.31) |
| U3 person-fit statistic | 0.89  (0.79, 0.99) | 1.08  (0.96, 1.20) | 1.11  (1.02, 1.21) | 1.14  (1.03, 1.25) |
| Extreme responses | 1.03  (0.92, 1.16) | 0.94  (0.84, 1.05) | 0.97  (0.89, 1.05) | 0.95  (0.85, 1.06) |

Note. Values are relative hazard ratios (95% confidence intervals) from within-study interaction models, meta-analyzed across studies. They indicate subgroup differences in the strength of associations between response-pattern indicators and incident dementia. Values below 1 indicate weaker associations in the focal subgroup relative to the reference subgroup; values above 1 indicate stronger associations. Models were adjusted for age, sex, and education and accounted for death as a competing risk.

# Supplementary Table 6. Study-level meta-regression testing whether response-pattern associations varied by number of survey scales

| **Survey response pattern index** | **Regression coefficient** | **95% confidence interval** |
| --- | --- | --- |
| Item non-response | 1.002 | (0.99, 1.02) |
| Random response errors | 1.006 | (0.98, 1.03) |
| Multivariate outlier responses | 0.998 | (0.98, 1.02) |
| Guttman errors | 1.003 | (0.98, 1.03) |
| *Lz* person-fit statistic | 1.001 | (0.98, 1.02) |
| *U3* person-fit statistic | 0.991 | (0.96, 1.02) |
| Extreme responses | 1.001 | (0.98, 1.02) |

*Note.* Values are exponentiated meta-regression slopes and 95% confidence intervals from study-level random-effects meta-regressions predicting study-specific log hazard ratios from the study-level mean number of survey scales used to calculate the response-pattern indices. Values above 1 indicate stronger associations in studies with more survey scales. Models used estimates adjusted for age, sex, and education to include all eight cohorts.

# References

Emons, W. H. (2008). Nonparametric person-fit analysis of polytomous item scores. *Applied Psychological Measurement, 32*, 224-247. https://doi.org/10.1177/0146621607302479

Falk, C. F., & Ju, U. (2020). Estimation of response styles using the multidimensional nominal response model: A tutorial and comparison with sum scores. *Frontiers in psychology, 11*, 72. https://doi.org/10.3389/fpsyg.2020.00072

Muthén, L. K., & Muthén, B. O. (2017). *Mplus: Statistical Analysis with Latent Variables: User's Guide (Version 8).* Los Angeles, CA: Muthén & Muthén.

Schneider, S. (2018). Extracting response style bias from measures of positive and negative affect in aging research. *The Journals of Gerontology, Series B: Psychological Sciences and Social Sciences, 73*, 64-74. https://doi.org/10.1093/geronb/gbw103

Tendeiro, J. N., Meijer, R. R., & Niessen, A. S. M. (2016). PerFit: An R package for person-fit analysis in IRT. *Journal of Statistical Software, 74*, 1-27. https://doi.org/10.18637/jss.v074.i05
